# Effect of Breed and Finishing Diet on Chemical Composition and Quality Parameters of Meat from Burguete and Jaca Navarra Foals

**DOI:** 10.3390/ani12050568

**Published:** 2022-02-24

**Authors:** Aurora Cittadini, María V. Sarriés, Rubén Domínguez, Mirian Pateiro, José M. Lorenzo

**Affiliations:** 1 Instituto de Innovación y Sostenibilidad en la Cadena Agroalimentaria (IS-FOOD), Universidad Pública de Navarra (UPNA), Campus de Arrosadia, 31006 Pamplona, Spain; aurora.cittadini@unavarra.es; 2 Centro Tecnológico de la Carne de Galicia, Avd. Galicia No. 4, Parque Tecnológico de Galicia, 32900 San Cibrao das Viñas, Spain; rubendominguez@ceteca.net (R.D.); mirianpateiro@ceteca.net (M.P.); 3 Área de Tecnoloxía dos Alimentos, Facultade de Ciencias, Universidade de Vigo, 32004 Ourense, Spain

**Keywords:** horsemeat, commercial feed, silage, organic diet, meat quality, sensory analysis

## Abstract

**Simple Summary:**

In modern society, the quality, the nutritional value and the environmental impact of foods are factors acquiring a relevant position from the consumers’ perspective. In this sense, horsemeat perfectly meets the current market requirements and is arousing great interest, since not only is it claimed as a healthier red meat alternative, but also due to its sustainable image. Nevertheless, it has been demonstrated that meat quality could be affected by several factors. In this sense, the purpose of our study is to assess the impact of breed (Jaca Navarra and Burguete) and type of finishing diet (conventional diet and silage with organic feed) on the composition and quality attributes of meat from Navarre endangered foals. Moreover, considering the shortage of information about the meat quality of these two autochthonous breeds, especially in the case of Jaca Navarra foals, this research could be considered a starting point to revalue and characterize these valuable equine breeds. Data showed that meat belonging to Burguete and traditional diet groups presented the highest intramuscular fat contents conferring ameliorate quality attributes, as marbling, tenderness and juiciness, which are some of the most required by meat consumers. Moreover, lower amounts of cholesterol were detected.

**Abstract:**

The purpose of this study was to investigate the influence of breed, Jaca Navarra (JN) vs. Burguete (BU), and finishing diet, conventional concentrate and straw, diet 1 (D1), vs. silage and organic feed, diet 2 (D2), on chemical composition and quality parameters of the *longissimus thoracis et lumborum* muscle from forty-six foals. Animals were reared under a semi-extensive system and slaughtered at a mean age of 21 months. The results reported that both studied effects had a significant (*p* < 0.05) impact on meat quality; however, it was the breed to strongly influence the majority of the parameters evaluated. In particular, BU foals reported the highest amounts of intramuscular fat, positively affecting the meat properties of marbling and texture traits. Moreover, this group presented higher values for L* and b* and the lowest cholesterol contents. As regards the diet, D1 increased the fat content in foals supplemented with this diet, improving the organoleptic properties of this group. On the other hand, the combination of silage and organic feed (D2) had an opposite trend. Thus, both BU and D1 groups presented enhanced quality attributes, such as marbling, juiciness and reduced hardness, which are some of the most demanded by meat consumers.

## 1. Introduction

The meat consumption pattern is globally changing, and the quality and sustainable image of meat are in the spotlight [1,2]. This trend is largely driven by an increasing consumer health-consciousness and environmental concern. As regards red meat, in fact, interest has been shifted towards alternative animal species, such as horse, among others [1]. In this sense, according to the data provided by the Food and Agriculture Organization of the United Nations (FAO), worldwide horse meat production is slowly rising, registering an increment of 7.57% between 2010 and 2020 (from 718,433 tons in 2010 to 772,829 tons in 2020) [3]. In particular, 55.58% of this production comes from Asia, the main producer at the world level and where this meat is also part of the traditional diet, especially in countries such as Kazakhstan [3,4]. As regards Europe, it produces 13.52%, occupying the third position of horse meat producers, after America (25.49%). In this context, Spain represents one of the European countries with the highest levels of production [3], and part of its production is exported to countries such as Italy, Belgium and France, where this meat is traditionally consumed [4,5,6]. However, in Spain, horse meat consumption is acquiring an important role [7,8]. Concretely, an important increment was observed in our country, where a total of 37,169 equids were slaughtered for meat production in 2020, obtaining 9601 tons of meat, 35.02% more than ten years earlier (7111 tons in 2010) [9]. Thus, these data could be indicative of the changes in consumer attitude towards this type of meat [10]. Furthermore, recent studies [5,11] confirmed that alternate red meats, such as equine meat, could be beneficial not only from a nutritional point of view but also at social, economic and environmental levels. Actually, horse meat was defined by some researchers as a “dietetic meat”, characterized by high-value proteins, iron and B-group vitamins, as well as low fat and cholesterol content and a favorable fatty acids profile, showing that its consumption may be favorable for human health [12,13,14,15,16,17]. Moreover, its productive chain had a low environmental impact and is sustainable from both an economic and social point of view. Indeed, it preserves the pasturage ecosystem, maintains population in rural areas, protects the area against fire and erosion, revitalizes deprived areas and stands out for its reduced methane and other greenhouse gases emission [5,18,19,20]. In addition, equid food production represents a relevant opportunity for valorizing animal biodiversity through the preservation and recovery of local breeds [5]. In this context, in the north of Navarre (NE Spain), two autochthonous and endangered foal breeds are located, “Jaca Navarra” (JN) and “Burguete” (BU), which, in the last decades, both the scientific community and farmers are trying to revalue and place in economic activities for meat production [2].

Nevertheless, considering the growing interest in horse meat features, a limited number of studies are available about these two breeds. Concretely, some works were realized about BU foals [7,10,21,22], but there is a dearth of knowledge about the quality and properties of JN foal meat.

Additionally, sensory characteristics play a crucial role in consumers’ satisfaction. Actually, sensorial properties related to texture, juiciness and flavor could have a relevant impact on eating quality and general acceptability [21,22]. Moreover, marbling, defined as the quantity and spatial distribution of visible white flecks of intramuscular fat (IMF) present within the lean in a muscle [23,24], is another remarkable parameter determining the quality of meat. It is closely associated with the palatability of cooked meat (such as tenderness, juiciness and flavor), and for this, meat with high marbling values is expected to have a better sensory quality [25]. However, also in this case, in equine, a scarce number of works has been published about these topics. To date, BU sensory profile and marbling traits have been investigated by some authors [26,27], while no information can be compiled for JN foals. In this context, several studies have observed that factors, such as breed [7,28] and finishing diet [14,29,30,31,32], among others, have a great effect on the meat characteristics of these monogastric animals.

In this sense, this investigation was carried out in order to give value to these two Navarre autochthonous breeds, as well as to fill the lack of information on meat quality of these two Navarre autochthonous breeds. Concretely, this study is aimed to evaluate the effect of breed (BU vs. JN) and finishing diet (conventional concentrates and straw—diet 1, vs. silage and organic feed—diet 2) on the physicochemical parameters, marbling traits, iron content and sensory profile of the *longissimus thoracis et lumborum* muscle from forty-six foals.

## 2. Materials and Methods

### 2.1. Animal Management and Sampling

Forty-six foals, twenty-seven from Jaca Navarra (JN) and nineteen from Burguete (BU) breeds, were employed in this experiment. Cittadini and coworkers [2] have previously provided a complete description of the animal management and experimental design. In short, animals obtained from local farms after weaning were reared under a semi-extensive livestock system. Actually, the herd was raised at pasture from 12 to 17 months of age. Successively, the animals were kept indoors in the experimental farm (Na 221 NA1) of the Institute for Agri-food and Technology and Infrastructures of Navarre (Roncesvalles, Navarre, Spain), where they were finished (during 3–4 months) with two different diets, named diet 1 (D1) and diet 2 (D2). A total of 22 foals (13 of JN and 9 of BU breeds), belonging to the D1 group, were fattened with conventional concentrates (starter and finisher ones) and straw (ad libitum). While 24 animals (14 of JN and 10 of BU breeds), the D2 group, were supplemented with silage (produced by local farmers) and an organic fodder (with certification of UE/no UE Organic Agriculture), where silage represented the majority component of the diet (approximately 60% of silage and 40% of organic feed). The chemical composition of feeds employed, shown in Table 1, was determined according to the Standard Official Methods of AOAC [33]. However, full information on their ingredients was recently reported [2,34].

An adaptation phase (14 days) to finishing diets was followed by the animals (using oats and silage) with the aim to avoid colics, which typically occur with an abrupt change in the alimentation. Thus, four separate groups of foals were formed: JN-D1, JN-D2, BU-D1 and BU-D2. The experimental procedures employed conform to the European Directive 2010/63/EU [35] and to the Spanish Royal Decree 53/2013 [36], which establish the base standards for the care and handling of research animals.

The equids (mean age of 21 months) were transported to an accredited abattoir (Protectora de carne S.L., Salinas de Pamplona, Navarre, Spain) the day before slaughter according to the European regulations [37], avoiding mixing groups and minimizing the stress of foals. After transport, animals were kept in livestock stores in separate pens and fasted (only water was available) for about 12 h prior to slaughter. The foals were stunned in the frontal region of the head with a captive bolt, slaughtered and dressed according to specifications described in the Council Regulation 1099/2009 [38]. Once sacrificed, the carcasses were allowed to chill for 24 h in a conventional room at 0 °C and the day after, the left half-carcasses were transported under refrigeration to the installations of Cárnicas Mutiloa (Sangüesa, Navarre, Spain) and stored in a chilling room (at 0 °C) for four days according to the local procedure. Successively, the *longissimus thoracis and lumborum* (LTL) muscle from each half-carcass was removed from the sixth to the thirteenth ribs and cut into 25 mm thick steaks, vacuum-packed and stored at −20 °C until subsequent analysis.

### 2.2. Chemical Composition

The moisture [39], protein [40] and ash [41] were determined in accordance with the International Standards Organization (ISO), while the approved procedure Am 5-04, established by the American Oil Chemistry Society [42], was employed to extract and quantify the intramuscular fat (IMF) content. Briefly, moisture percentage was obtained by weight loss experimented by the sample (5 g) maintained in the oven (Memmert UFP 600, Schwabach, Germany) at 105 °C, until constant weight. For IMF content determination, 1 g of sample was submitted to a liquid/solid extraction employing petroleum ether in an extractor apparatus (Ankom^HCI^ Hydrolysis System XT10, Macedon, NY, USA) at 90 °C for 60 min [42]. IMF was calculated based on gravimetric difference. Protein content was determined according to the Kjeldahl method, multiplying the total nitrogen content by a conversion factor of 6.25. A sample (0.5 g) was subjected to a reaction with sulfuric acid (Kjeldahl tablets, containing 99.9% of potassium sulfate and 0.1% of selenium, were employed as a catalyst) in a digester (Büchi Digest System K-437 with Scrubber B-414, Flawil, Switzerland). Organic nitrogen was transformed into ammonium sulfate, which was distilled in alkaline conditions in a distillation apparatus (Büchi Distillation Unit B-324, Flawil, Switzerland). Ash percentage was obtained calculating the weight loss experienced by 3 g of sample maintained in a muffle furnace (Carbolite^®^ RWF 12–13, Hope Valley, UK) in a porcelain capsule at 600 °C until constant weight.

### 2.3. Analysis of Cholesterol

For determination of total cholesterol, saponification, extraction and identification were carried out according to the protocol described by [43]. Briefly, for saponification, 2 g of homogenized sample was placed in a screw Teflon-lined cap tube, and 0.25 g of L-ascorbic acid and 5 mL of saponification solution were added. The air was removed from the reaction by displacement with nitrogen gas, and the sample was vortexed until the ascorbic acid was completely dissolved. The samples were rested for 5 min and then were shaken. The saponification was performed in a shaking water bath (THER-SPIN, Orto Alresa, Madrid, Spain) (200 rpm) at 85 °C for 45 min. The samples were vortexed after 20 min of the saponification process. After saponification, the samples were cooled and, successively, 1.5 mL of distilled water and 3 mL of 25 μg/mL BHT solution in n-hexane were added. At this point, the samples were vigorously vortexed and centrifuged at 1500× *g* for 3 min, in order to accelerate phase separation. An aliquot of the upper layer (n-hexane) was transferred into a small screw Teflon-lined cap tube, and a spatle tip of anhydrous sodium sulfate was added. Finally, the tube was briefly shaken, and an aliquot of the n-hexane layer was filtered through a 0.45-μm nylon syringe filter (Filter Lab, Barcelona, Spain) into an amber screw-cap vial with Teflon septum. The HPLC systems used were an Alliance 2695 model (Waters, Milford, MA, USA) and a 996 Photodiode Array Detector (Waters, Milford, MA, USA). Empower 3TM advanced software (Waters, Milford, MA, USA) was employed in order to control system operation and result management. The analysis of cholesterol was carried out using a normal-phase silica column (SunFireTM Prep Silica, 4.6 mm ID × 250 mm, 5 μm particle size; Waters, Milford, MA, USA), with Photodiode Array detection for cholesterol (208 nm). The solvent (2% *v*/*v* isopropanol in n-hexane) flow rate was 1 mL/min; the run lasted for 17 min, and the temperature of the column oven was adjusted to 30 °C. The content of total cholesterol in foal meat was quantified based on the external standard technique from a standard curve of peak area vs. concentration. The results were expressed as mg of cholesterol/100 g of meat.

### 2.4. Physicochemical Analysis

The pH of the samples was determined using a digital pH-meter (Hanna Instruments, Eibar, Spain) equipped with a penetration glass probe. At the beginning of the analysis, the device was calibrated using buffers with pH values 4.00 and 7.00 (Scharlab, S.L., Sentmenat, Spain). Meat color parameters (L*—brightness, a*—greenness/redness and b*—blueness/yellowness) were measured in the CIELAB space [44] employing a portable colorimeter (Konica Minolta CM-600d, Osaka, Japan) with the following settings: pulsed xenon arc lamp, standard illumination D65, angle of 10° viewing angle geometry and aperture size of 8 mm. Data acquisition was carried out using the color data software SpectraMagic^TM^ Nx CM-S100w 2.90.0007 (Konica Minolta, Osaka, Japan). Before measurements, the colorimeter calibration was carried out with a white ceramic tile according to the manufacturer’s recommendations. For each sample, three measurements were performed in three homogeneous and representative areas, free of intramuscular fat. Samples were allowed to bloom for 30 min before measuring. The relative content of myoglobin (Mb), metmyoglobin (MMb) and oxymyoglobin (OMb) was obtained by recording the reflex attenuance of incident light at four different isosbestic points 572, 525, 473 and 730 nm [45].

The water holding capacity (WHC) was measured in two ways: pressure and cooking loss. WHC calculated as pressure loss was determined as suggested by Grau and Hamm [46] and modified by Sierra [47]. Briefly, 5 g (W_i_) of minced sample was collocated between two filter papers (Whatman, 90 mm Ø, Maidstone, UK). Successively, the filter papers with the sample were located between two thin glass plates and subjected to constant pressure by a weight of 2250 g for 5 min. Afterward, the meat was removed from the paper, and the weight (W_f_) of the meat was recorded. The WHC was expressed as a percentage of pressure loss, obtained by the difference in weight between W_f_ and W_i_. For cooking loss determination, steaks were weight and placed in vacuum package bags and cooked in a water bath with automatic temperature control (JP Selecta, Precisdg, Barcelona, Spain) until they reached an internal temperature of 70 °C, monitored by thermocouples type K (Comark, PK23M, St Neots, UK) connected to a data logger (Comark Dilligence EVG, N3014). Once cooked, the samples were cooled at room temperature (for about 30 min), and the percentage of cooking loss was calculated by the difference in weight between the cooked and raw samples. Successively, three pieces of meat of 10 × 10 × 25 mm^3^ (height × width × length) were removed parallel to the muscle fiber direction from each cooked sample and used for the Warner Blatzler (WB) shear force test. All samples were cut perpendicular to the muscle fiber direction at a crosshead speed of 3.33 mm/s using a texture analyzer (TA.XTplus, Stable Micro Systems, Vienna Court, Godalming, UK). The samples were totally cut employing a Warner Bratzler shear blade with a triangular slot cutting edge (1 mm thickness). The maximum shear force, shown by the highest peak of the force-time curve, corresponds to the maximum resistance of the sample to be cut.

### 2.5. Computer Image Analysis (CIA)

**Figure 1:**
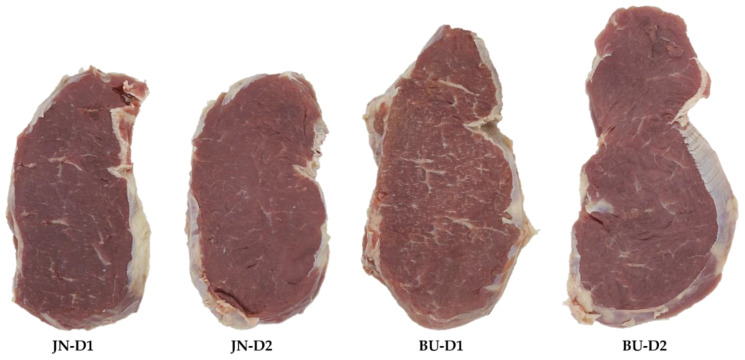
Appearance of the steaks of the *longissimus thoracis et lumborum* muscle. JN = Jaca Navarra, BU = Burguete, D1 (Diet 1) = conventional concentrate + straw, D2 (Diet 2) = silage + organic concentrate.

The image analysis is considered a reliable, nondestructive and objective approach for marbling assessment [48]. Image capture and computer image analysis (CIA) technology measurements were employed in order to assess marbling parameters and color values of foal meat. Figure 1 shows the appearance of the steaks employed for this analysis.

In particular, the digital image of the two faces of each steak was obtained using a Digieye^®^ Version 2.81 image system (VeriVide Ltd., Leicester, UK), which consists of a Nikon D90 digital camera connected to a controlled lighting cabin (VeriVide Ltd., Leincester, UK) and a computer provided with appropriate software (DigiPix^®^, DigiPix Technologies, Bengalore, India). Prior to image capture, the machine was calibrated with a white uniformity board and successively with a color chart (DigiTizer) in accordance with the manufacturer’s recommendations. Moreover, each steak was allowed to bloom for 30 min before the image acquisition. The captured images were saved as 4288 × 2848 pixels TIF format and, a 15 cm ruler was employed as a scale in order to obtain the pixel and consequently the mm ratio for the successive image analysis. Actually, digital images were later processed using ImageJ software (ImageJ, 1.52t, U.S. National Institutes of Health, Bethesda, MD, USA; http://imagej.nih.gov/ij/ accessed on 1 November 2021) according to the methodology established by Mendizabal et al. [49] to obtain the following measures: the *longissimus thoracis et lumborum* muscle area, number of marbling flecks, average size of marbling flecks, marbling area, marbling percentage (defined as the ratio between the marbling area and muscle area, multiplying for 100) and the RGB color. In detail, considering the latter variable, “R” stands for red color, “B” means blue and “G” stands for green. The color range varies from 0 to 225. The value “0” means that 100% of the light is reflected, and the value “225” means that 100% of the light is absorbed. The average red, green and blue values were obtained. All image acquisitions and measurements were realized by a single experienced operator with the purpose of eliminating subjective operator differences.

### 2.6. Iron Determination

For the analysis of iron content, the procedure previously described by Lorenzo et al. was followed [50] with modifications. In brief, the ash, obtained as described in Section 2.2, was dissolved in 10 mL of 1 M HNO_3_ and then filtered through filter paper (Filter-lab, 110 mm Ø, Filtro Anoia, S.A., Barcelona, Spain). The quantification of Fe^+^ (selected wavelength 239.5 nm) was performed by inductively coupled plasma-optical emission spectroscopy (ICP-OES), using a Thermo-Fisher ICAP 6000 plasma emission spectrometer (Thermo-Fisher, Cambridge, UK), equipped with a radio frequency source of 27.12 MHz, a peristaltic pump, a spraying chamber and a concentric spray nebulizer, completely controlled by ICP software using 99.996% liquid argon plasma gas (Praxair, Madrid, Spain). An external standard for setting the calibration curve was employed in order to determine the Fe^+^ content. The final value was obtained by calculating the average of three determinations and was expressed as mg/100 g of meat.

### 2.7. Sensorial Analysis

The sensory analysis was carried out according to the ISO 13299: 2017 regulation [51] in order to define the sensorial profile of the four treatments studied (BU-D1, BU-D2, JN-D1 and JN-D2). The evaluation was performed in the sensorial analysis laboratory of the Public University of Navarre equipped with individual cabinets in line with the UNE-EN ISO 8589:2010/A1:2014 regulation [52]. Tasting was performed under red light in order to avoid color bias since appearance characteristics of samples are not variables to evaluate in the present study [53]. Furthermore, considering the pandemic state and its consequent restrictions (July 2020), it followed a safety and hygienic protocol approved by the Department of occupational health of the University. Thus, in order to carry out this study, a quantitative descriptive analysis (QDA) was conducted with a panel composed of seven panelists (with ages between 35 and 55 and from both genders) selected from the Public University of Navarre (Pamplona, Spain) staff. This test is one of the most complete methods used for sensory characterization since it can provide a complete description of the organoleptic properties [54]. Tasters were firstly trained in accordance with the methodology described by UNE-EN ISO 8586:2014 [55] with the attributes and the scale to use during the three sessions.

The steaks were cooked in a 180 °C pre-heated electric grill (Jata GM3000, Tudela, Spain) inside aluminum foil until they reached an internal temperature of 70 °C [56], which was measured by a portable probe thermometer (HI-98501, Hanna instruments, Eibar, Spain). Each steak was cut into pieces (10 × 10 × 25 mm), wrapped in aluminum foil and kept in a warmer provided with sand until the analysis was performed. Moreover, the samples were coded with a randomized 3-digit number, and the tasting order was designed and indicated to the tasters in order to avoid first sample and carry-over effects [57]. A total of nine sessions were carried out, and each panelist tasted the four samples (BU-D1, BU-D2, JN-D1 and JN-D2) in each session following a balanced block completed experimental design. Members of the panel were provided with water and unsalted toasted bread to clean the palate and remove residual flavors at the beginning of the session and between samples. In particular, the following attributes were evaluated: odor intensity and liver odor, flavor intensity, metallic flavor and sweet taste, hardness, juiciness and fibrousness. The panelists evaluated these attributes using a structured scale, where 0 represented “absence/the lowest intensity of the attribute” (left side) and 10 was “the highest intensity of the attribute” (right side).

### 2.8. Statistical Analysis

The SPSS statistical software (SPSS 25.0, Chicago, IL, USA) was employed to perform all statistical analyses. Firstly, Shapiro–Wilk and Levene tests were applied in order to verify the normal distribution and variance homogeneity, respectively. Afterward, an analysis of variance (ANOVA) with the general linear model (GLM) procedure was carried out on composition, physicochemical traits, marbling values and iron content. The model used was:

Y_ij_ = μ + B_i_ + FD_j_ + (B × FD)_ij_ + ε_ij_ (1)

where Y_ij_ is the observation of dependent variables, μ the overall mean, B_i_ the effect of breed, FD_j_ the effect of finishing diet, B × FD is the effect of interaction of the ith breed and the jth finishing diet and ε_ij_ the residual random error associated with the observation. The interaction, B × FD, was included in the model only when significance was shown. In all analyses, Duncan’s *t*-test was used to compare least-squares mean and to evaluate in detail among which of the four studied groups are present significant differences. Significance was indicated at *p* < 0.05. In addition, correlations between variables (*p* < 0.05) were determined by correlation analyses using Pearson’s linear correlation coefficient. Finally, a factorial analysis of the parameters studied with significant differences (*p* < 0.05) among treatments was carried out in order to evaluate the relationship between the main variables. Principal components analysis (PCA) was used as an extraction method and performed on the correlation matrix. Prior to carrying out PCA, the Kaiser-Mayer-Olkin (KMO) measure of sample adequacy and the Bartlett test of sphericity were conducted.

## 3. Results and Discussion

### 3.1. Effect of Breed and Finishing Diet on Chemical Composition

The chemical composition of foal meat is shown in Table 2. Data indicated that moisture and intramuscular fat values were significantly (*p* < 0.001) affected by both of the studied factors, while ash contents were influenced (*p* < 0.001) only by the breed.

Moisture content has a relevant influence on meat quality attributes, such as juiciness, tenderness, among others [58]. In particular, the outcomes obtained for moisture were higher (*p* < 0.001) in JN foals and in animals fed with D2. As regards the breed, the JN group recorded a mean percentage higher than those found in 8–14 month-old Catria horses [59] and in older Jeju foals (30–36 months old) [60,61]. On the other hand, the BU group reported amounts similar to those found in 16 month-old female foals of the same breed [26]. However, considering the finishing diet, our results are in agreement with the range of values (68.34–75.43%) described in the literature for foals fattened with a finishing diet [7,14,26,28,29,30,31,59,60,61,62,63,64,65,66]. Additionally, moisture was negatively correlated with IMF contents (r= −0.820, *p* < 0.001), showing that there is an inverse relationship between these two variables.

Considering the IMF amounts, in fact, the highest (*p* < 0.001) percentages were detected in BU and D1 foals, which reported the lowest moisture contents. According to the breed, on average, IMF accounted for 5.01% in BU foals, while JN presented a mean of 3.79%. In contrast, previous studies [7,29,32] did not observe a significant effect of breed on the IMF content in foal meat. Our outcomes for the BU group are close to the values obtained by Sarriés et al. [26] in 24-month-old foals of the same breed reared under a semi-extensive system. Whereas, although JN values are not similar to those obtained from other foals studied previously, our results fit with the range of values (2.08–5.27%) found in the literature [58,61], eliminating extreme IMF percentages (the highest and lowest). However, these outcomes could be expected considering the different carcass traits and morphology of these two autochthonous breeds. Actually, Cittadini et al. [2] has recently observed that BU foals presented a major adiposity in comparison to JN foals. As regards the type of diet, unsurprisingly, the D1 group presented greater values (*p* < 0.001) than the D2 one. The composition of these diets [2] could justify our outcomes since feeds employed in the traditional finishing diet (D1) had a higher fat percentage than silage and the organic feed included in D2. Furthermore, the D1 group obtained amounts in agreement with some authors [26], who investigated 24-month-old BU foals finished with concentrates for 7–8 months. However, most of the investigations [7,14,29,30,31,32,59,63] on finished-foals reported values lower than ours. In particular, our outcomes were much higher than those obtained by some authors [14,32,33], who studied the meat quality from GM foals and crossed GM × HB and GM × BU foals finished with concentrate at pasture for 3–4 months. Similarly, Franco and coworkers [30], investigating GM foals finished indoors for 3 months, showed values much lower than ours. In the same way, lower values were also noticed in other studies [7,30,60,63], where foals (11–24 months old) from different breeds were fattened during distinct finishing periods (from 2 to 7 months). These discrepancies are normal and could be explicated by several factors, such as the different breed and slaughter age, the diverse finishing conditions, including the duration of the supplementation time, fattening at pasture or indoors, composition, quantity and type of feeds employed, among others. Nevertheless, it is evident that the type of diet had a relevant effect on this parameter, as previously observed in the literature [30,31,32,65].

On the other hand, as regards protein content, no significant differences (*p* > 0.05) were detected among the studied groups. Our values are in agreement with those published in previous works for BU [26], HB [63] and Catria horses [59]. Conversely, recent studies [17,30,31,62], studying foals from other breeds fattened during different periods (3–26 months), reported amounts higher than ours. These findings could be related to the distinct characteristics of feeds used, the duration of the finishing period, as well as to the differences in slaughter age (9–32 months old) and breed (IHDH, GM and Jeju foals) between the studies, among other factors. Moreover, it is worth noting that our outcomes are also in contrast with those obtained from foals fattened at pasture [14,32,33] and managed under an extensive livestock system [31,33,66], which recorded greater values than ours. This could be related to the different anatomical modifications affecting the muscles in animals with a major movement rate as horses finished at pasture or in freedom extensive systems, which brings to an increment of protein amounts in meat [58]. Muscle actually presents different biological and physiological features for its mechanical function in live horses, providing consequently distinct chemical composition and properties [67].

Regarding the ash content, JN animals showed greater percentages (*p* < 0.001) than BU ones, recording, on average, values of 1.21% vs. 1.11%, respectively. This difference could be related to a distinct mineral content among groups. Outcomes obtained from JN foals are close to those found by Franco et al. [65] and Juárez et al. [7], studying concentrate-finished Galician Mountain × Hispano Bretón (GM × HB) (1.5 kg of fodder/foal-day) and HB foals, respectively. Meanwhile, the BU group presented amounts in line with previous investigations [7,68] on equids of the same breed. On the contrary, similar values were found among the D1 and D2 groups, showing percentages (a mean of 1.17%) in agreement with those observed in previous studies [14,64] of concentrate-finished foals.

Hence, considering the four treatments, data reflected the results above commented. Samples belonging to the BU-D1 group recorded the lowest moisture values and, as expected, the highest IMF percentage (5.81%), while JN-D2 foals showed the opposite behavior. On the whole, the proximate composition was unaffected (*p* > 0.05) by the interaction of breed and type of diet.

**Figure 2:**
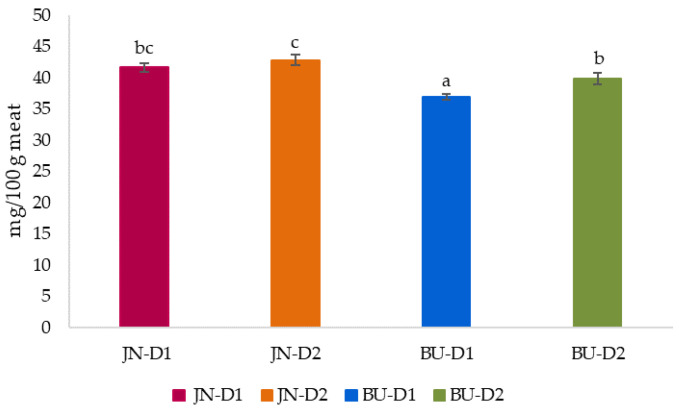
Effect of breed and finishing diet on cholesterol content (expressed as mg/100 g meat) of the *longissimus thoracis et lumborum* muscle of foals. ^a–c^ Mean values with different letter differ significantly (*p* < 0.05; Duncan test); JN = Jaca Navarra, BU = Burguete, D1 (Diet 1) = conventional concentrate + straw, D2 (Diet 2) = silage + organic concentrate.

Figure 2 shows the results for cholesterol content in the *longissimus thoracis and lumborum* muscle of foals. Cholesterol represents an important component of cell membranes, brain and steroidogenic tissues [69]. It is well-known that its high intake and content in animal food products could be related to an elevated risk of cardiovascular diseases, such as coronary heart disease and high blood pressure, as well as diabetes [70,71]. As regards our results, statistical analysis indicated that data were influenced (*p* < 0.05) by both studied factors.

In fact, significant (*p* < 0.05) differences were detected among groups, where JN and D2 foals showed higher amounts than their respective counterparts (42.24 vs. 38.46 mg/100 g meat for JN and BU groups; 41.57 vs. 39.70 mg/100 g meat for D2 and D1, respectively). These findings are in agreement with the literature [14,72], confirming that samples with elevated IMF contents show proportionately fewer membrane polar lipids and, consequently, lower amounts of the cholesterol associated with these membranes, as in our case. Actually, a negative correlation between cholesterol and IMF contents was detected (r= −0.685, *p* < 0.001). In comparison with previous works, our values are much lower than those found in GM foals reared at pasture [66,73] and GM × HB finished with a conventional concentrate [32], ranging from 60 to 64 mg/100 g meat. Whereas JN foals, in particular the JN-D2 group, obtained values closed to those showed by Dominguez et al. [14], studying 26-month-old GM × BU foals finished with commercial concentrate. On the other hand, the same foals (GM × BU) presented lower values than ours when they were supplemented with linseed-rich concentrate. Thus, these findings highlighted and confirmed that breed and especially the diet could highly influence the cholesterol values.

Moreover, though the 2015–2020 Dietary Guidelines for Americans did not include the recommendations of limiting the daily cholesterol intake to the maximum of 300 mg/day, the Guidelines still advise consuming dietary cholesterol as low as possible [71]. Nowadays, attention is moved from fat quantity to fat quality and to lower consumption of dietary saturated fatty acids and total cholesterol [74,75,76]. Therefore, considering our outcomes, according to a daily consumption of 150 g of steak, trimmed of all visible fats, except for intramuscular fat, our samples represent a cholesterol intake of about 61.02 mg, which corresponds to approximately 20% of the recommended maximum daily cholesterol intake. This aspect explicates as horsemeat consumption could have a key role in health safeguard and in the prevention of nutrition-derived human diseases [58]. Thus, as discussed above, JN-D2 represented the group with the highest cholesterol values, followed by JN-D1, BU-D2 and BU-D1 (JN-D2 < JN-D1 < BU-D2 < BU-D1). Any significant interactions between the main categories (B × FD) were found.

### 3.2. Effect of Breed and Finishing Diet on Physicochemical Parameters

Table 3 shows the physicochemical parameters of the *longissimus thoracis et lumborum* muscle of our samples. Statistical analysis found that breed had a significant (*p* < 0.05) influence on physicochemical parameters. Actually, all studied variables were affected by breed apart from a* values. On the contrary, the data were unaffected (*p* > 0.05) by the type of diet, except for pH, cooking loss and shear force values.

Regarding pH, both studied effects had an influence on this variable. Actually, the JN group reported significantly (*p* < 0.01) higher values than the BU one. Meanwhile, considering the diet, samples belonging to the D1 group showed greater (*p* < 0.05) values than those from the D2 group. Our outcomes disagree with those described by other authors [7,29,32], who did not observe breed effects on pH values of foal meat. On the other hand, Dominguez et al. [14] and Lorenzo et al. [32], investigating GM × BU and GM × HB foals, confirmed that the type of finishing diet and livestock system could have a significant influence on the pH of equine meat. According to the literature [62,63,77], differences in this variable could also be related to other factors, such as distinct physical activity, levels of glycogen stored, free glucose and lactic acid or different pre-slaughter state of the animals (as stress degree and starvation duration). Considering the four groups, BU-D2 showed the lowest values in comparison with the others ones. [14]. Additionally, this variable was also affected by the interaction B × FD. Actually, within the BU foals, a change of tendency was observed, JN-D1, JN-D2 and BU-D1 groups reported similar values, while in the BU foals fed with the D2 diet, a reduction of pH values was observed. Knowing that pre-slaughter and *post-mortem* conditions of animals were the same, it could be supposed that in this breed (BU), the type of alimentation could be the reason for this outcome in the BU-D2 group. However, our results are within the acceptable range (<6) [14].

As regards color parameters, significant differences (*p* > 0.05) were not found between diet groups. This finding is in contrast with the literature, where different authors [14,32,33,65] detected a significant effect of diet on meat colorimetric characteristics. Whereas, a trend similar to ours was reported by Franco et al. [30], who investigated GM foals reared under two different livestock systems, semi-extensive vs. freedom extensive systems. Nonetheless, it was observed a clear “breed effect” (*p* < 0.001) on lightness (L*) and yellowness (b*) indices, where the BU group showed higher values than the JN one. These results could also be related to the higher quantity of IMF found in the BU group, which could favor the increase of these two variables [10,62,68]. In addition, a positive and significant correlation between IMF and L* was detected (r = 0.449, *p* < 0.01), and b* values (r = 0.448, *p* < 0.01). Considering the scientific literature, Juárez et al. [7], studying BU and HB foals, also found that muscle color traits were affected by breed. However, BU foals recorded a mean L* value (39.68) greater than those found in the same breed in previous studies [7,10]. On the whole, our samples, both the BU and JN ones, were more luminous in comparison with those from other concentrate-finished foal breeds as Italian Heavy Draft horses (IHDH) [17], GM [31], HB [7], Jeju [61] and cross-breeding as GM × BU [64] and GM × HB (supplemented with 1.5 kg/day of concentrate) [65]. A similar tendency was observed for the b* parameter. Actually, also, in this case, our data (for both breeds) showed higher values than those found by other authors [7,10,14,28,30,31,32,61,65], studying color traits in foal meat. In contrast, the BU and JN groups reported similar values (*p* > 0.05) for a*. Our outcomes for BU foals are close to those obtained by Sarriés et al. [10], studying 24-month-old BU animals. Nevertheless, our samples showed a lower red hue in comparison with previous studies [12,60,62,64]. Moreover, the BU-D1 samples represented the group with the highest L* and b* values, while the JN-D2 group included darker samples and with a lower yellow hue. However, any significant interactions (*p* > 0.05) between the main categories (B × FD) were reported.

In relation to pigment forms, statistical analysis indicated that breed had a significant (*p* < 0.05) influence on these variables, while similar (*p* > 0.05) values were observed among diet groups. Myoglobin is a water-soluble protein, and its content in meat plays a key role since it determines meat color via its chemical forms [77]. In particular, in equine meat, it has been noted that an increased affinity of oxygen to combine with the bright red oxymyoglobin and convert to brown metmyoglobin [58]. Considering myoglobin content, the JN group showed higher (*p* < 0.001) values in comparison with its counterpart (22.08% vs. 17.21%, respectively). The physical activity of the animal could change the metabolic characteristics of the muscles, which might be associated with the existing differences in the myoglobin content [78]. Furthermore, animal temperament also could be the cause of different Mb contents [12]. Our results disagree with previous studies [14,64], where higher values than ours were observed. With reference to MMb and OMb, conversely, BU foals presented the greatest (*p* < 0.05) values in comparison with JN ones. In the case of OMb, this trend could be correlated with b* index. Indeed, a positive correlation was found between these two variables (r = 0.486, *p* < 0.01), being in line with the data mentioned above. However, considering MMb and OMb values, divergent results were published in the literature. Actually, Ruiz et al. [64] reported values higher than ours, while Dominguez et al. [14] observed lower values. Therefore, considering the four treatments, the JN-D2 group reported the greatest levels of Mb followed by JN-D1, BU-D1 and BU-D2. However, considering MMb and OMb variables, BU-D1 and BU-D2 showed the highest percentages, respectively. Significant interactions among the studied effects were observed for these parameters, except for MMb.

Water holding capacity (WHC) and texture parameters play a relevant role in the consumer decision since they provide information about meat quality, in particular in terms of juiciness and tenderness [32]. Our results showed that cooking loss and shear force were affected (*p* < 0.001) by both studied effects, while press loss was significantly (*p* < 0.01) influenced only by the breed. Regarding WHC, it is worth highlighting that this variable is also affected, among several other factors, by the raw meat composition, particularly by the content and distribution of IMF. The presence of IMF, in fact, decreases the moisture diffusivity coefficient [79]. Actually, statistical analysis showed a negative and significant correlation between WHC variables and IMF content (r = −0.393, *p* < 0.01 for press loss and r = −0.535, *p* < 0.001 for cooking loss). This behavior is reflected in our results, where the group with the highest IMF content, BU foals, reported the lowest (*p* < 0.05) values of WHC in comparison with the JN group. Following the same trend, D1 samples presented significantly lower (*p* < 0.05) percentages for cooking loss. Our outcomes are not consistent with those published by some authors [30,31,32,63,65], who studied finished-foals and observed lower values than ours. Meanwhile, Seong et al. [61] reported values greater than those found in BU foals but lower than those from JN foals.

As regards texture, foals belonging to the BU and D1 groups also showed the lowest (*p* < 0.001) values for shear force in comparison with their counterparts. This finding is in line with the literature [14,30,31,62], confirming that differences in texture parameters could be associated with the breed and feeding conditions, among other factors. In addition, our results for the BU and D1 samples were lower than those found in other equines supplemented with concentrates [10,29,32,61,63,64]. However, our outcomes could be related to the higher IMF contents of BU and D1 foals, which could be responsible for the improved tenderness of samples [30]. This behavior is also supported by the presence of a negative and significant correlation among the shear force and IMF values (r = −0.668, *p* < 0.001). Moreover, our findings are also in line with the results obtained for WHC parameters, where samples with a higher water loss (JN and D2) were also the toughest. In addition, statistical analysis confirmed that shear force and WHC values were positively correlated (r = 0.396, *p* < 0.01 for press loss and r = 0.615, *p* < 0.001 for cooking loss). Considering the four groups, in agreement with the above discussed, foals presenting the lowest values for WHC and texture were those belonging to the BU-D1 group, followed by BU-D2, JN-D1 and JN-D2 (BU-D1 < BU-D2 < JN-D1 < JN-D2). Thus, according to the tenderness classification devised by Belew et al. [80], the BU-D1 and BU-D2 samples can be defined as “very tender” (WB shear force < 31.4 N cm^−2^), while JN-D1 and JN-D2 as “tender” (31.4 N cm^−2^ < WB shear force < 38.3 N cm^−2^). Texture parameters confirmed that horsemeat is a tender meat in comparison with other species [5]. However, the interactions between breed and finishing diet were not significant for all variables, apart from shear force.

### 3.3. Effect of Breed and Finishing Diet on Marbling and Color Parameters Determined by CIA

The data obtained by CIA are reported in Table 4. Marbling parameters were significantly affected (*p* < 0.05) by both investigated factors, except for muscle area, influenced only by the breed. This trend is consistent with the literature, where some investigators [25,81] stated that meat marbling could be affected by breed and diet, among others.

In particular, BU and D1 foals showed the highest (*p* < 0.05) values for the number of marbling flecks, average particle size and marbling area and percentage. These outcomes could be justified by their composition; actually, BU and D1 foals represented the groups with also the highest IMF percentages. These results could be expected since it is recognized that meat marbling is directly related to the IMF content [25]. Indeed, marbling parameters were positively correlated with IMF content (r = 0.460, r = 0.516, r = 0.640 and r = 0.681, *p* < 0.05 for number of marbling flecks, average particle size and marbling area and percentage, respectively). Considering muscle area, also in this case, the BU group showed higher (*p* < 0.01) values than JN foals. This finding could be related to the different sizes and characteristics as weight at slaughter of these two Navarre horses, where the BU breed showed to be predominant (539.11 vs. 414.33 kg for BU and JN foals, respectively) [2]. Our outcomes for muscle area, number of flecks and marbling area were higher than those found by Sarriés et al. [82]. Nevertheless, BU foals showed average size particles values closer to those found by the abovementioned authors. Meanwhile, marbling percentages of our samples are within the range of values (0.6–6.8%) obtained by the same investigators [82].

Considering the RGB color parameters, the values were affected only by the breed, apart from red. In fact, red values showed similar (*p* > 0.05) values among groups coinciding with a* outcomes (Table 2). As regards green and blue values, the BU group reported the majority (*p* < 0.05) values in comparison with JN foals. Conversely, according to the diet groups, RGB values did not present significant differences (*p* > 0.05). However, our outcomes are not consistent with previous studies since our data were higher than those reported for BU foals by Sarriés et al. [82]. While Ruiz et al. [64], investigating GM × BU foals, showed values greater than ours, except for green (85.9), which was lower than those obtained for BU animals in our study.

On the whole, CIA outcomes were in line with the data obtained for IMF and color commented in the previous sections. BU-D1 represented the group with the highest values for marbling, as could be expected. In addition, it also recorded the greatest values for green and blue colors. However, no interactions (B × FD) were found (*p* > 0.05) on any of the parameters evaluated, except for muscle area, marbling area and percentage.

### 3.4. Effect of Breed and Finishing Diet on Iron Content

**Figure 3:**
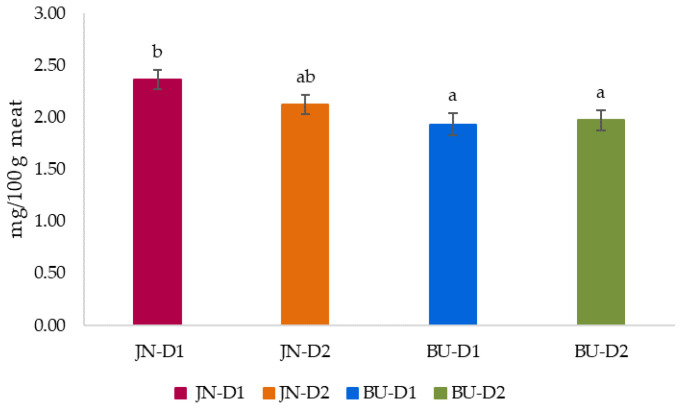
Effect of breed and finishing diet on iron content (expressed as mg/100 g meat) of the *longissimus thoracis et lumborum* muscle of foals. ^a,b^ Mean values with different letter differ significantly (*p* < 0.05; Duncan test); JN = Jaca Navarra, BU = Burguete, D1 (Diet 1) = conventional concentrate + straw, D2 (Diet 2) = silage + organic concentrate.

The influence of breed and finishing diet on iron content is illustrated in Figure 3. Statistical analysis indicated that the breed significantly (*p* < 0.01) affected Fe^+^ values of the *longissimus thoracis et lumborum* muscle. On the other hand, the type of finishing diet had not effect (*p* > 0.05) on the iron content of our samples, showing similar values among groups.

In relation to the breed, JN foals recorded a greater iron amounts than BU ones, an average of 2.23 vs. 1.95 mg/100 g meat, respectively. Our outcomes are comparable with data found in the literature for horse meat, ranging from 1.95 to 4.58 [58,59]. BU foals, in particular, presented values approximated to those found in animals from the same breed [68] and in Catria Horses [59]. Horse meat is recognized as an important source of iron in comparison with chicken (0.9 mg/100 g), pork (1.4 mg/100 g), lamb (2.23 mg/100 g) and beef (2.6 mg/100 g) meat [11,83]. Nonetheless, in this study, both breeds only showed higher values than those observed in chicken and pork meat. Considering the four treatments, the JN-D1 group was the group with the highest amounts of iron among the studied groups. In addition, also in comparison with the values obtained from other species [11,83], meat from the JN-D1 group could be considered rich in iron, while BU-D1 showed the lowest values. However, iron contents were unaffected (*p* > 0.05) by the interaction of breed and type of diet.

### 3.5. Sensory Profile of Foal Meat

**Figure 4:**
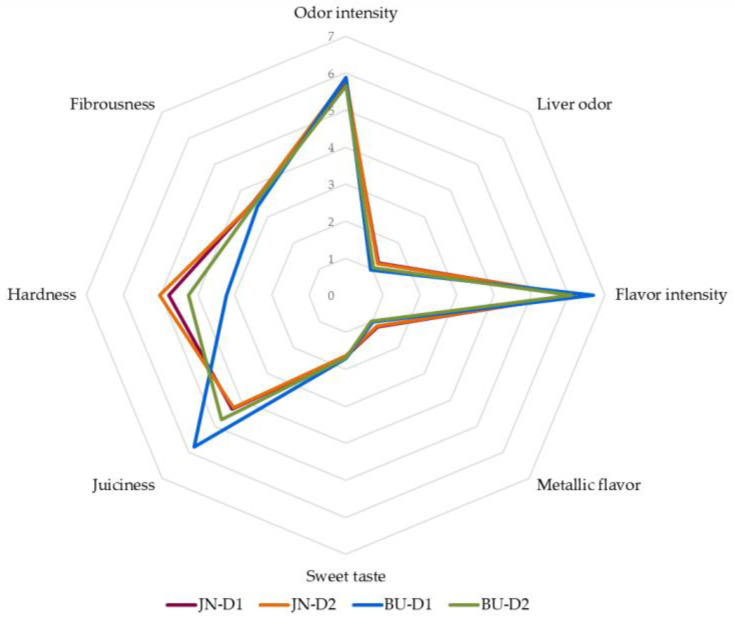
Means values for the sensorial traits of the *longissimus thoracis et lumborum* muscle of foals. JN = Jaca Navarra, BU = Burguete, D1 (Diet 1) = conventional concentrate + straw, D2 (Diet 2) = silage + organic concentrate.

Mean scores for the sensory characteristics are shown in Figure 4. In detail, JN samples reached a mean score of 5.77 for odor intensity, while panelists were able to detect a slight liver odor (1.22). As regards flavor attributes, this group presented a moderate flavor intensity (6.17) and a very low metallic flavor (1.19). Tasters also distinguished a delicately sweet taste (1.64) for JN samples. Moreover, this group was characterized by an intermediate level of hardness (4.90) and slightly low scores for juiciness (4.31) and for fibrousness (3.54). The BU group reported a moderate odor intensity (5.77) and very low liver odor (1.00). The intensity of flavor detected recorded a mean score of 6.38, while a very slight metallic flavor (1.00) was perceived. In addition, in this group, a mild sweet taste was also detected (1.7). In relation to texture parameters, BU samples were described by the panelists as a meat mildly juicy (5.26), not tough and fibrous (3.74 and 3.44, respectively).

Considering the type of diet, samples belonging to the D1 group presented a moderate odor intensity and minimum liver odor. Flavor intensity recorded an average score of 6.42 and 1.12 for metallic flavor. A delicate sweet taste (1.68) was also reported for D1 samples. Additionally, the D1 group was scored by the panel with intermediate values for juiciness (5.06), slightly low values for hardness (4.00) and fibrousness (3.46). Similarly, the D2 group reported a moderate intense odor and flavor, while liver odor, metallic flavor and sweet taste were slightly perceived. As regards texture, D2 meat did not record high values for juiciness (4.51), a moderate score for hardness (4.64) and not excessive punctuation for fibrousness (3.51).

On the whole, considering the four groups, it is worth highlighting that the BU-D1 group was defined by the panel as tender and juicy, while the JN-D2 group reached an intermediate score for hardness and low for juiciness. These results agree with data obtained from the instrumental analysis described previously. Moreover, as commented above, texture parameters are closely related to IMF contents [30]. In this study, significant correlations were found between sensory texture parameters and meat quality traits. Actually, IMF content was negatively correlated with hardness (r = −0.407, *p* < 0.01). Unsurprisingly, also with marbling features, as marbling percentage reported a negative correlation with hardness (r = −0.496, *p* < 0.001) and positive with juiciness (r = 0.449, *p* < 0.01). Moreover, data obtained from the WB test agree with those reported from the sensory test. Shear force was positively correlated with hardness (r = 0.546, *p* < 0.001) and negatively with juiciness (r = −0.415, *p* < 0.01). As commented above, the cooking loss is strictly related to juiciness, indeed a negative correlation was found among these two parameters (r = −0.509, *p* < 0.001). Actually, sensory analysis confirmed that samples with the highest IMF contents, marbling percentages and the lowest WB shear force and cooking loss values reported lower hardness and higher juiciness scores, as in the case of the BU-D1 group. Meanwhile, JN-D2 foals showed intermediate values for hardness and low scores for juiciness, in line with their IMF contents and shear force outcomes.

Furthermore, in comparison with the limited number of studies on sensory characteristics of foal meat, our results for flavor intensity, metallic flavor and sweet taste present a similar trend to those reported by other authors [31]. In contrast, previous studies [21,26] showed scores for sweet taste higher than ours. This parameter was considered as an indicator of pasture flavor [26,84]; thus, these differences could be related to the distinct periods of grazing of animals. On the other hand, our samples showed an odor slightly less intense than foals studied by Franco et al. [31]. Meanwhile, the BU-D1 group stood out for its low hardness and high juiciness in comparison with data observed in previous works [21,23,32,54]. Moreover, previous investigations [31,53] reported higher values for fibrousness compared to our data. Nevertheless, the shortage of sensory studies regarding foal meat, as well as the use of different evaluation scales, make it complicated to establish comparisons with other works. On the whole, considering the profile obtained, it may be hypothesized that our findings agree with other authors [31,53], who observed that genotype, as well as the type of diet, have minor influence on the sensory traits of foal meat. The statistical analysis of the described variables is provided in Supplementary Table S1.

### 3.6. Principal Components Analysis

Principal component analysis allows obtaining a better overall idea of the relationship among the studied variables. In detail, the PCA showed that about 72.56% of the variability was explained by the three principal components. The correlation matrix for this model presented a determinant close to zerom indicating the existence of significant correlations between variables and the suitability of this type of analysis (Table 5).

**Figure 5:**
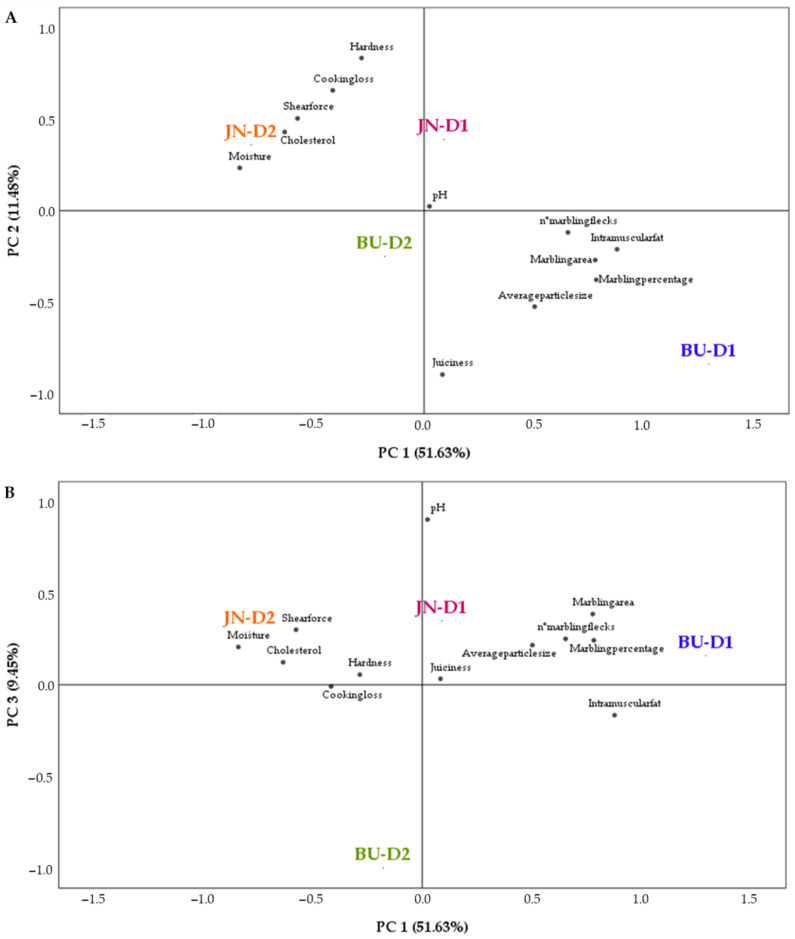
Relationships among the treatments and the main quality attributes obtained by PCA. (**A**) Projection of the variables and treatments in the plane defined by the first two principal components. (**B**) Projection of the variables in the plane defined by PCs one and three.

Figure 5 illustrates the results obtained by PCA analysis. The principal component 1 (PC 1) was the most important variable in terms of differences among treatments (B × FD) as it accounted for 51.63% of the total variability. As it can be seen in Figure 5A, PC 1 was positively related with intramuscular fat and marbling parameters (number of marbling flecks, marbling area and marbling percentage), whereas it had a negative relation with moisture, shear force and cholesterol. Moreover, the BU-D1 group had the greatest component 1 value. Therefore, this group was related to IMF and marbling traits, while it showed a negative link with moisture, shear force and cholesterol. As shown, samples from BU-D1 and JN-D1 are on the positive side of PC 1, while BU-D2 and JN-D2 are on its negative side. Therefore, PC 1 was able to discriminate among diet groups. On the other hand, the principal component 2 (11.48%) was positively related to hardness and cooking loss and negatively linked to juiciness. Foal steaks from BU-D1 and BU-D2 were located on the negative PC2 axis whereas, JN-D1 and JN-D2 samples were on the positive PC2 axis. Thus, JN-D2 samples were strongly related to hardness and cooking loss and negatively correlated with juiciness. A similar trend was followed by the JN-D1 group. The PC 2 so was able to differentiate between breeds. Finally, the principal component 3 (9.45%) was positively linked to pH. As can be seen in Figure 5B, both the JN groups and BU-D1 are on the positive side of PC 3, while BU-D2 is on the negative side. Hence, JN samples and BU-D1 are positively related with pH. Hence, this analysis confirmed the outcomes obtained in the previous sections.

## 4. Conclusions

The results obtained showed that meat quality was affected by both studied effects; however, it was the breed that influenced the majority of the parameters evaluated. Concretely, BU foals reported the highest values of intramuscular fat, positively affecting meat properties as marbling and texture traits, both at instrumental and sensorial levels. On the other hand, JN foals reported the opposite trend. Nevertheless, it is worth noting that D1 significantly increased the IMF content of the foals supplemented with this diet, enhancing the organoleptic properties of this group. Hence, meat belonging to the BU and D1 groups presented ameliorated quality attributes, such as marbling, juiciness and tenderness, which could increase consumers’ acceptability. Overall, although the differences observed among groups, meat obtained from the four treatments can be considered tender and is characterized by a low cholesterol level. In this sense, promising results were obtained and are in line with the demand of meat consumers. Our outcomes confirm that horse meat could serve as a suitable meat source for consumers seeking an alternative to traditional red meats.

However, considering the potential of these two breeds, further investigations could be planned to improve the production system and to enhance the meat quality of these Navarre autochthonous foals, especially of JN animals, and to satisfy the current consumer requirements. Otherwise, taking into consideration the shortage of information about foal meat, particularly in the case of the Jaca Navarra breed, this research could be considered a starting point to promote and characterize these valuable endangered foal breeds.

## Figures and Tables

**Table 1:** Chemical composition of oats, concentrates, straw and silage allocated to the foals.

Parameters	Oats	Starter Feed	Finisher Feed	Straw	Silage	Organic Feed
Moisture (%)	11.64	-	-	-	-	-
Fat (%)	5.58	3.40	6.00	1.95	3.71	4.80
Protein (%)	8.78	12.90	12.80	2.00	16.22	8.50
Ash (%)	2.59	8.50	4.50	3.88	8.81	5.00
Crude fiber (%)	12.90	14.00	10.40	-	-	7.00
Starch (%)	40.00	-	-	-	-	-
Phosphorus (%)	0.26	-	-	-	-	-
Calcium (%)	0.08	-	-	-	-	-
Sodium (%)	-	1.00	0.30	-	-	0.30
Methionine (%)	-	-	-	-	-	0.20
Lysine (%)	-	-	-	-	-	0.30
Dry matter (%)	-	-	-	-	77.90	-
Acid detergent fiber (%)	-	-	-	-	26.50	-
Neutral detergent fiber (%)	-	-	-	-	56.30	-

**Table 2:** Effect of breed and finishing diet on the chemical composition of the *longissimus thoracis et lumborum* muscle of foals.

Parameters	JN		BU			Sig.		
	D1	D2	D1	D2	SEM	B	FD	B × FD
Moisture (%)	71.20 ^b^	72.22 ^c^	69.91 ^a^	71.51 ^bc^	0.178	***	***	ns
Intramuscular fat (%)	4.24 ^b^	3.36 ^a^	5.81 ^c^	4.30 ^b^	0.169	***	***	ns
Protein (%)	20.01	20.01	20.34	20.30	0.131	ns	ns	ns
Ash (%)	1.22 ^b^	1.19 ^b^	1.12 ^a^	1.11 ^a^	0.011	***	ns	ns

^a–c^ Mean values in the same row (corresponding to the same parameter) with different letters differ significantly (*p* < 0.05; Duncan test); SEM: Standard error of the mean; Sig.: significance: *** (*p* < 0.001, ns. (not significant); JN = Jaca Navarra, BU = Burguete, D1 (Diet 1) = conventional concentrate + straw, D2 (Diet 2) = silage + organic concentrate; B = Breed; FD = Finishing diet.

**Table 3:** Effect of breed and finishing diet on physicochemical properties of the *longissimus thoracis et lumborum* muscle of foals.

Parameters	JN		BU			Sig.		
	D1	D2	D1	D2	SEM	B	FD	B × FD
pH	5.59 ^b^	5.59 ^b^	5.59 ^b^	5.49 ^a^	0.011	**	*	**
Color parameters								
L*	37.54 ^a^	37.38 ^a^	40.47 ^b^	38.97 ^ab^	0.345	***	ns	ns
a*	13.91	13.94	14.07	14.26	0.147	ns	ns	ns
b*	12.43 ^a^	12.10 ^a^	13.76 ^b^	13.52 ^b^	0.163	***	ns	ns
Pigment form								
Myoglobin (%)	20.11 ^b^	23.92 ^c^	18.99 ^b^	15.60 ^a^	0.597	***	ns	***
Metmyoglobin (%)	26.80 ^ab^	25.21 ^a^	27.52 ^b^	27.12 ^b^	0.323	*	ns	ns
Oxymyoglobin (%)	52.24 ^ab^	50.75 ^a^	54.05 ^bc^	55.83 ^c^	0.473	***	ns	*
Water holding activity								
Pressure loss (%)	16.23 ^ab^	16.71 ^b^	14.67 ^a^	14.76 ^a^	0.294	**	ns	ns
Cooking loss (%)	26.63 ^bc^	28.21 ^c^	22.68 ^a^	24.72 ^b^	0.443	***	*	ns
Texture parameters								
Shear force (N cm^−2^)	34.71 ^c^	36.98 ^c^	22.65 ^a^	30.52 ^b^	1.008	***	***	*

^a–c^ Mean values in the same row (corresponding to the same parameter) with different letter differ significantly (*p* < 0.05; Duncan test); SEM: Standard error of the mean; Sig.: significance: *** (*p* < 0.001), ** (*p* < 0.01), * (*p* < 0.05), ns. (not significant); JN = Jaca Navarra, BU = Burguete, D1 (Diet 1) = conventional concentrate + straw, D2 (Diet 2) = silage + organic concentrate; B = Breed; FD = Finishing diet.

**Table 4:** Effect of breed and finishing diet on marbling characteristics and color (determined by CIA) of the *longissimus thoracis et lumborum* muscle of foals.

Parameters	JN		BU			Sig.		
	D1	D2	D1	D2	SEM	B	FD	B × FD
Marbling traits								
Muscle area (cm^2^)	116.57 ^ab^	109.77 ^a^	119.92 ^bc^	126.54 ^c^	1.714	**	ns	*
N° marbling flecks	49.50 ^a^	43.23 ^a^	56.36 ^b^	47.89 ^a^	1.226	*	**	ns
Average particle size (mm^2^)	5.89 ^ab^	5.16 ^a^	6.90 ^b^	5.91 ^ab^	0.186	*	*	ns
Marbling area (cm^2^)	2.95 ^b^	2.33 ^a^	3.89 ^c^	2.43 ^ab^	0.128	*	***	*
Marbling percentage (%)	2.35 ^a^	1.97 ^a^	3.66 ^b^	2.07 ^a^	0.112	***	***	***
RGB values								
Red	114.69	115.04	115.70	116.01	0.390	ns	ns	ns
Green	83.40 ^a^	82.88 ^a^	87.36 ^b^	86.54 ^b^	0.576	***	ns	ns
Blue	82.75 ^ab^	82.34 ^a^	85.74 ^b^	85.23 ^ab^	0.545	**	ns	ns

^a–c^ Mean values in the same row (corresponding to the same parameter) with different letter differ significantly (*p* < 0.05; Duncan test); SEM: Standard error of the mean; Sig.: significance: *** (*p* < 0.001), ** (*p* < 0.01), * (*p* < 0.05), ns. (not significant); JN = Jaca Navarra, BU = Burguete, D1 (Diet 1) = conventional concentrate + straw, D2 (Diet 2) = silage + organic concentrate; B = Breed; FD = Finishing diet.

**Table 5:** Factor loadings for the main parameter studied on the first three principal components obtained.

Parameters	PC 1	PC 2	PC 3	Communalities
Juiciness	0.083	−0.896	0.033	0.812
Hardness	−0.286	0.835	0.056	0.783
Moisture	−0.843	0.234	0.207	0.808
Intramuscular fat	0.881	−0.211	−0.166	0.849
n° marbling flecks	0.657	−0.119	0.251	0.509
Marbling area	0.781	−0.270	0.387	0.833
Marbling percentage	0.786	−0.378	0.243	0.821
Average particle size	0.505	−0.525	0.217	0.577
pH	0.024	0.024	0.904	0.819
Cooking loss	−0.418	0.658	−0.009	0.608
Shear force	−0.579	0.505	0.301	0.681
Cholesterol	−0.638	0.431	0.123	0.608
Percent of variance	51.630	11.479	9.448	
Cumulative percentage	51.630	63.109	72.556	

## Data Availability

Not applicable.

## Supplementary Materials

The following supporting information can be downloaded at: https://www.mdpi.com/article/10.3390/ani12050568/s1, Table S1: Effect of breed and finishing diet on the sensorial traits of the *longissimus thoracis et lumborum* muscle of foals.
