# Supplementary material for: Effect of Breed and Finishing Diet on Chemical Composition and Quality Parameters of Meat from Burguete and Jaca Navarra Foals
Source: Animals (Basel). 2022 Feb 24;12(5):568. doi: 10.3390/ani12050568 (PMC8908835; doi:10.3390/ani12050568)
Supplement: Supplementary file 1 [file animals-12-00568-s001.zip › animals-1576657-supplementary.pdf]

animals MDPI

Table S1. Effect of breed and finishing diet on the sensorial traits of *longissimus thoracis et lumborum* muscle of foals.

	JN		BU			Sig.		
	**D1**	**D2**	**D1**	**D2**	**SEM**	**B**	**FD**	**B×FD**
Olor Intensity	5.70[a]	5.84[a]	5.89[a]	5.64[a]	0.115	ns	ns	ns
Liver Olor	1.24[b]	1.20[ab]	0.96[a]	1.05[ab]	0.044	*	ns	ns
Flavor Intensity	6.15[ab]	6.20[ab]	6.68[b]	6.08[a]	0.094	ns	ns	ns
Metallic flavor	1.21[a]	1.18[a]	1.03[a]	0.98[a]	0.043	*	ns	ns
Sweet taste	1.65[a]	1.63[a]	1.71[a]	1.68[a]	0.079	ns	ns	ns
Juiciness	4.34[a]	4.29[a]	5.79[b]	4.74[a]	0.117	***	*	*
Hardness	4.79[bc]	5.02[c]	3.22[a]	4.25[b]	0.115	***	**	ns
Fibrousness	3.55[a]	3.53[a]	3.38[a]	3.50[a]	0.119	ns	ns	ns

[a-c] Mean values in the same row (corresponding to the same parameter) with different letter differ significantly ($p < 0.05$; Duncan test); SEM: Standard error of the mean; Sig.: significance: *** ($p < 0.001$), ** ($p < 0.01$), * ($p < 0.05$), ns. (not significant); JN = Jaca Navarra, BU = Burguete, D1 (Diet 1) = conventional concentrate + straw, D2 (Diet 2) = Silage + organic concentrate; B = Breed; FD = Finishing diet.
